# Acute effects of mixed circuit training on hemodynamic and cardiac autonomic control in chronic hemiparetic stroke patients: A randomized controlled crossover trial

**DOI:** 10.3389/fphys.2022.902903

**Published:** 2022-07-19

**Authors:** Guilherme F. Fonseca, Adrian W. Midgley, Sandra A. Billinger, André C. Michalski, Victor A. B. Costa, Walace Monteiro, Paulo Farinatti, Felipe A. Cunha

**Affiliations:** ^1^ Laboratory of Physical Activity and Health Promotion, Graduate Program in Exercise Science and Sports, University of Rio de Janeiro State, Rio de Janeiro, Brazil; ^2^ Department of Sport and Physical Activity, Edge Hill University, Ormskirk, Lancashire, United Kingdom; ^3^ Department of Neurology at University of Kansas Medical Center, Kansas City, MO, United States; ^4^ KU Alzheimer’s Disease Center, Fairway, KS, United States

**Keywords:** autonomic nervous system, blood pressure, circuit-based exercise, exercise, rehabilitation

## Abstract

**Objectives:** To investigate whether a single bout of mixed circuit training (MCT) can elicit acute blood pressure (BP) reduction in chronic hemiparetic stroke patients, a phenomenon also known as post-exercise hypotension (PEH).

**Methods:** Seven participants (58 ± 12 years) performed a non-exercise control session (CTL) and a single bout of MCT on separate days and in a randomized counterbalanced order. The MCT included 10 exercises with 3 sets of 15-repetition maximum per exercise, with each set interspersed with 45 s of walking. Systolic (SBP) and diastolic (DBP) blood pressure, mean arterial pressure (MAP), cardiac output (Q), systemic vascular resistance (SVR), baroreflex sensitivity (BRS), and heart rate variability (HRV) were assessed 10 min before and 40 min after CTL and MCT. BP and HRV were also measured during an ambulatory 24-h recovery period.

**Results:** Compared to CTL, SBP (∆-22%), DBP (∆-28%), SVR (∆-43%), BRS (∆-63%), and parasympathetic activity (HF; high-frequency component: ∆-63%) were reduced during 40 min post-MCT (*p* < 0.05), while Q (∆35%), sympathetic activity (LF; low-frequency component: ∆139%) and sympathovagal balance (LF:HF ratio: ∆145%) were higher (*p* < 0.001). In the first 10 h of ambulatory assessment, SBP (∆-7%), MAP (∆-6%), and HF (∆-26%) remained lowered, and LF (∆11%) and LF:HF ratio (∆13%) remained elevated post-MCT *vs.* CTL (*p* < 0.05).

**Conclusion:** A single bout of MCT elicited prolonged PEH in chronic hemiparetic stroke patients. This occurred concurrently with increased sympathovagal balance and lowered SVR, suggesting vasodilation capacity is a major determinant of PEH in these patients. This clinical trial was registered in the Brazilian Clinical Trials Registry (RBR-5dn5zd), available at https://ensaiosclinicos.gov.br/rg/RBR-5dn5zd.

**Clinical Trial Registration:** https://ensaiosclinicos.gov.br/rg/RBR-5dn5zd, identifier RBR-5dn5zd

## 1 Introduction

Stroke is one of the leading causes of death and disability worldwide (GBD, 2019). Indeed, 17% of stroke cases lead to death within 5 years and the rate of recurrence is as high as 41% at 28 days after the first episode (Chen et al., 2020). Among risk factors associated with cerebrovascular disease, hypertension is regarded as the most important and is the most prevalent in stroke survivors (Pistoia et al., 2015). It is suggested that maintaining blood pressure (BP) levels to <150/90 mmHg reduces the risk of stroke (James et al., 2014). Engagement in regular physical exercise is an important tool for controlling the risk of stroke due to elevated BP (Chong and Sacco, 2005). The 5.1 and 2.5 mmHg reduction in systolic (SBP) and diastolic blood pressure (DBP), respectively, observed in stroke populations in response to an exercise intervention (Wang et al., 2019) is similar to the 4.3 and 2.5 mmHg reductions from antihypertensive therapy (Liu et al., 2009). This highlights the importance of physical exercise as a non-pharmacological approach to post-stroke management of BP. A meta-analysis of 18 cohort studies and 5 case-control studies concluded that the risk of incidence of stroke and associated mortality is 27% lower in high *vs.* low active individuals (Lee et al., 2003). In addition, regular exercise promotes improvements in cardiorespiratory fitness and muscle strength, such as increased peak oxygen uptake (VO_2peak_), exercise tolerance, walking ability, gait speed/endurance, stair climbing and self-efficacy (Brogardh and Lexell, 2012).

Some studies have demonstrated that aerobic (Liu et al., 2012) and resistance (Dos Santos et al., 2014) exercise may promote acute and chronic reductions in BP in apparently healthy individuals, with the magnitude of acute reductions being predictive of the magnitude of chronic reductions. This acute reduction in BP in response to exercise bouts is commonly referred to as post-exercise hypotension (PEH) (Pescatello et al., 2004). However, little is known about this phenomenon in post-stroke individuals. In a pioneering study involving seven post-stroke individuals aged 56 ± 10 years, Lai et al. (2015) observed that a single bout of aquatic walking (15 min at 70% VO_2peak_) induced PEH for 9 h compared to a non-exercise control session (CTL). Although a novel finding of this study was that stroke patients may experience PEH, ambulatory blood pressure monitoring (ABPM) was limited to 12 h. Notably, assessment of 24-h and nighttime BP have been considered optimal to estimate cardiovascular risk. The higher the 24-h and nighttime BP, the greater the risk of death and cardiac events (Yang et al., 2019). Moreover, 24-h BP is more predictive of target organ damage and mortality outcomes than office BP measurements (Grassi et al., 2013; Redon, 2013). We are not, however, aware of any study that has investigated BP during the 24 h after exercise in a stroke population.

Aerobic exercise is widely recommended for the management of BP (Ghadieh and Saab, 2015). In the context of stroke rehabilitation, resistance exercise has been recommended for increasing the independence of hemiparetic individuals during activities of daily living, increasing gait velocity, and improving functional mobility by the development of upper and lower limb strength (Billinger et al., 2014). However, little information exists on the effectiveness of resistance exercise performed in isolation or combined with aerobic exercise in the management of BP in chronic post-stroke patients. A recent meta-analysis (Saunders et al., 2020) evaluating the effects of different exercise modalities on health outcomes in post-stroke patients reported that none of the 75 reviewed studies investigated the impact of resistance exercise on BP. Despite this lack of empirical evidence, the most recent joint guidelines for the management of stroke survivors from the American Heart Association and American Stroke Association recommended that stroke patients should perform mixed circuit training (MCT) at least 3 days/week (Billinger et al., 2014). It is worth noting that MCT seems to be effective in inducing PEH in individuals with no history of stroke (Brown et al., 1994; Paoli et al., 2013), but whether similar BP responses occur in post-stroke patients is yet to be determined.

To the best of our knowledge, only the study of Lai et al. (2015) has investigated the acute ambulatory BP responses to exercise in post-stroke patients. Although evidencing that this population may exhibit PEH, the authors did not investigate its underlying mechanisms. During recovery from exercise, lowered BP results from reductions in cardiac output (Q), systemic vascular resistance (SVR), or both (Chen and Bonham, 2010). Although the mechanistic basis of PEH has not been fully elucidated, current evidence suggests that autonomic control, commonly measured by heart rate variability (HRV) (Task-Force, 1996), may play an important role *via* changes in sympathetic activity and baroreflex resetting (Halliwill et al., 2013). Nevertheless, controversies surround this topic, with some studies observing increased sympathetic activity and autonomic balance concomitant to PEH (Teixeira et al., 2011; Cunha et al., 2015; Cunha et al., 2016; Fonseca et al., 2018), whereas others observed increased parasympathetic activity (Park et al., 2006) or even no changes in autonomic control (Park et al., 2008; Anunciação et al., 2016).

All these findings are from populations with no history of stroke and may not be generalized to post-stroke patients, which often show severe autonomic dysfunction (McLaren et al., 2005). To date, limited data from post-stroke populations are available regarding acute autonomic responses to exercise and its possible role in eliciting PEH. Francica et al. (2015) observed decreased sympathovagal balance after exercise compared to baseline in post-stroke patients, but this blunted response in cardiac autonomic control was not associated with PEH. Moreover, the 20-min assessment period during post-exercise recovery was short and the exercise involved a submaximal test rather than bouts consistent with physical activity guidelines for stroke survivors. It is therefore important to investigate the relationship between BP and cardiac autonomic control after more ecologically valid bouts of exercise, such as MCT.

Thus, the primary aim of this study was to investigate whether a single bout of MCT can elicit PEH in chronic hemiparetic stroke patients. A secondary aim was to investigate possible mechanisms underlying acute BP changes during two recovery conditions: (i) 40-min of laboratory-phase monitoring of SBP, DBP, MAP, Q, SVR, HRV, and baroreflex sensitivity (BRS); and (ii) 24-h of ambulatory-phase monitoring of SBP, DBP, MAP, and HRV. We hypothesized that, compared to CTL, a single bout of MCT would be able to elicit PEH in chronic post-stroke patients in parallel with reductions in SVR and followed by a compensatory increase in sympathovagal balance.

## 2 Methods

### 2.1 Ethical approval

This study was approved by the Ethics Committee at the University of Rio de Janeiro State (CAAE: 07618118.4.0000.5259) and registered in the Brazilian Clinical Trials Registry (RBR-5dn5zd). The experimental procedures were conducted in line with ethical guidelines from the Declaration of Helsinki. Participants were informed of the requirements, as well as the benefits and risks associated with participation in this study, and subsequently provided written informed consent.

### 2.2 Participants

Potentially eligible participants were recruited from the University Hospital Pedro Ernesto and Piquet Carneiro Polyclinic of the State University of Rio de Janeiro. The following inclusion criteria were applied: i) chronic stroke (≥ 6 months) with right or left hemiparesis; ii) ability to walk without supervision; iii) enrolled in a neuro-motor rehabilitation program for at least 6 months; iv) a score above 36 on the Berg balance scale; v) a minimum score of 50 on the Fugl-Meyer scale; and vi) not dieting or exhibiting any extreme dietary habits, including disinhibited and restrained eating tendencies. Exclusion criteria included: i) current smoker; ii) uncontrolled hypertension; iii) patients with clinical manifestations of hyper or hypothyroidism; iv) acute or chronic hepatic disease; v) patients with a history of angina or tests compatible with myocardial ischemia; vi) previous history of acute myocardial infarction and/or myocardial revascularization; vii) clinical signs of heart failure, symptomatic cardiac arrhythmia, or clinically significant valve disease; viii) history of drug or alcohol abuse; ix) history of psychiatric or neurological disease other than stroke; and x) severe aphasia or a cognitive-communication deficit.

**FIGURE 1:**
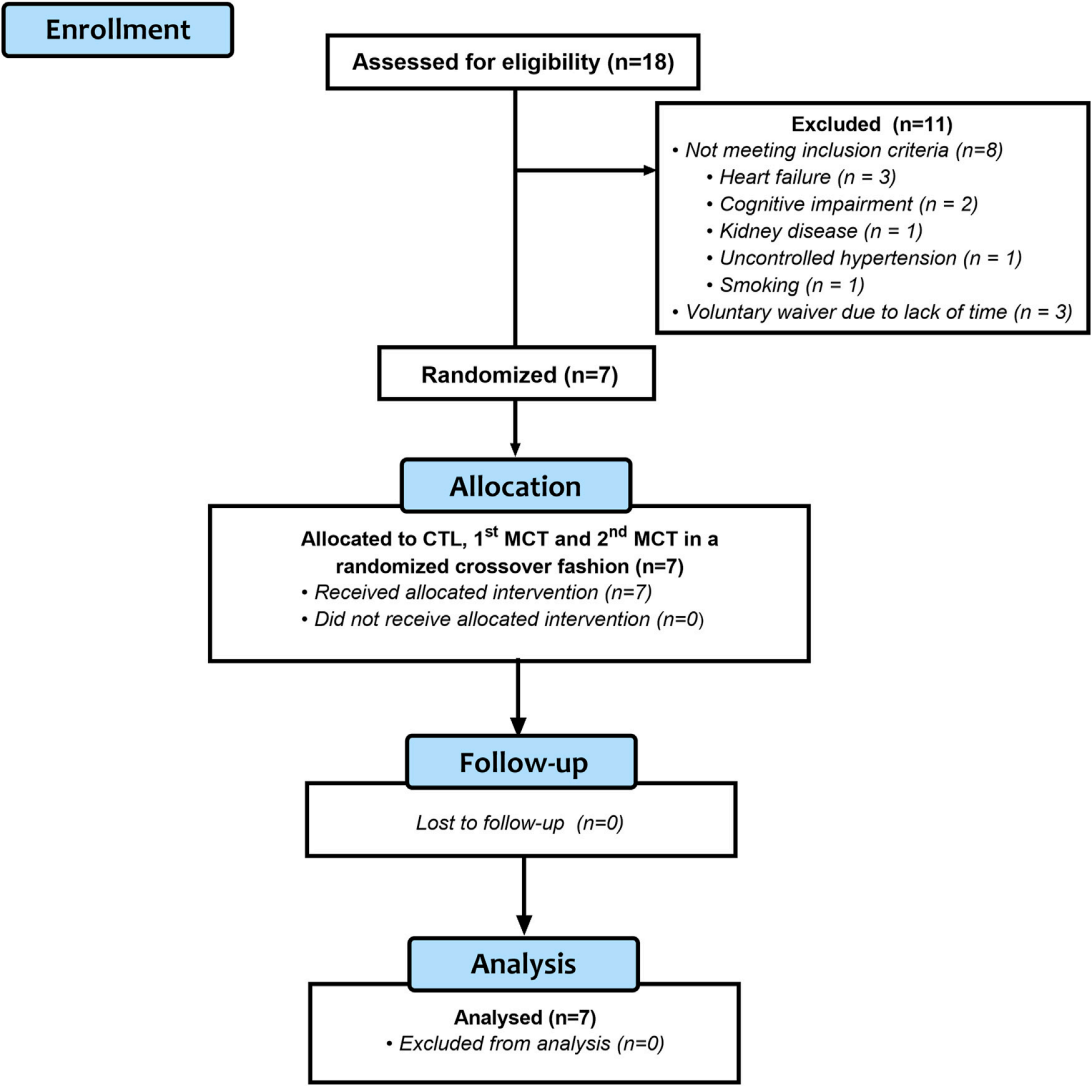
Flowchart for the study. MCT, mixed circuit training; CTL, non-exercise control session.

Eighteen individuals initially volunteered to participate in the study, with 11 of these excluded due to the presence of the following: heart failure (*n* = 3), cognitive impairment (*n* = 2), kidney disease (*n* = 1), uncontrolled hypertension (*n* = 1), smoking (*n* = 1), and voluntary waiver due to lack of time (*n* = 3). Seven participants (4 males and 3 females; 4 with right and 3 with left hemiparesis) were therefore considered eligible after the initial screening. Figure 1 shows the flowchart for the study.

### 2.3 Experimental design

**FIGURE 2:**
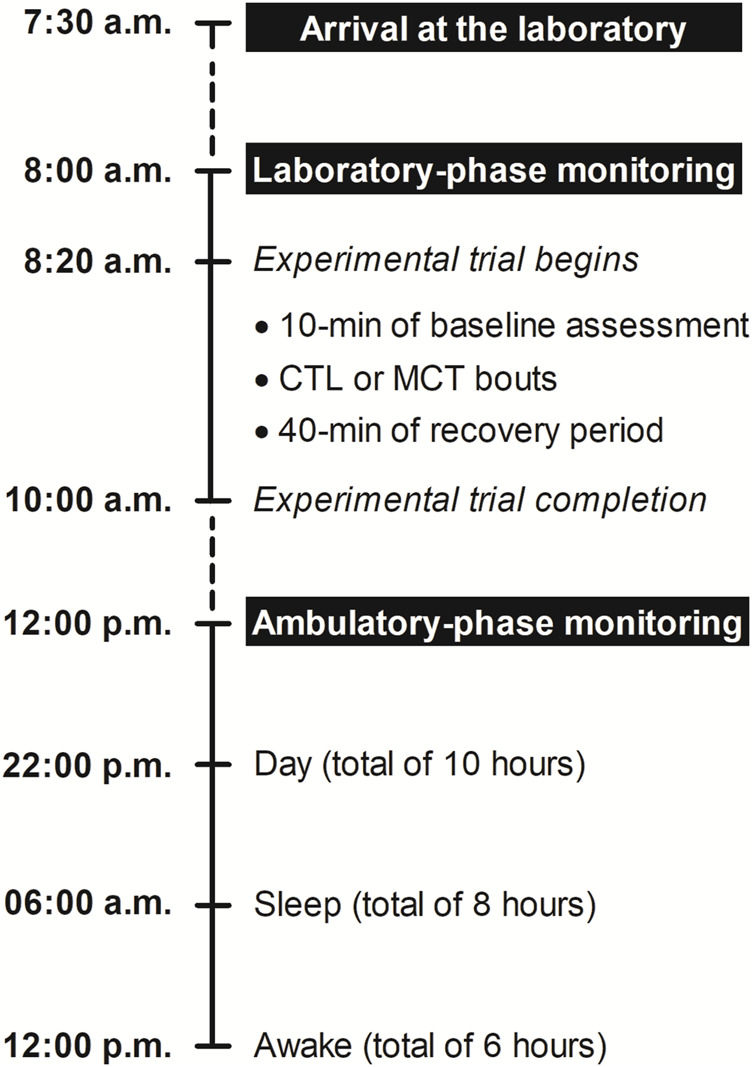
Overview of the study design. CTL, non-exercise control session; MCT, mixed circuit training.

Before the experimental trials, participants visited the laboratory over 3 weeks to undergo health screening, preliminary measurements (i.e., anthropometry, functional motor performance, cognitive status, and resting hemodynamic and cardiac autonomic control), familiarization with the exercises included in the MCT, and assessment of test-retest reliability for 15-repetition maximum (15-RM) tests. Figure 2 illustrates the timeline of assessments performed during each of the two experimental trials. The study incorporated a computer-generated randomized, controlled crossover design, including non-exercise control (CTL) and MCT sessions separated by 48–72 h. The randomization was conducted using Microsoft™ Excel software, which generated and assigned random numbers to the intervention and CTL. Randomization was performed by ACM and the statistical analyses by FAC. CTL and MCT took place in a thermoneutral environment (i.e., 21°C–24°C and relative humidity between 50%–70%) and always in the morning (8–10 a.m.) to negate the effects of circadian BP variations. In the 24 h preceding the experimental trials, participants were instructed to avoid any physical exercise, and abstain from alcohol, soft drinks, or caffeine. The laboratory-phase monitoring consisted of measuring BP, hemodynamic, and autonomic outcomes immediately before and for 40 min after CTL and MCT. After this, patients had the ambulatory monitoring device fitted. In this ambulatory-phase monitoring, BP and HRV were assessed for 24 h.

### 2.4 Procedures

#### 2.4.1 Anthropometric assessments

Body mass was assessed using a digital balance scale with a maximal capacity of 150 kg and accuracy of 0.1 kg (Welmy™, São Paulo, Brazil). Height was measured with a graded stadiometer with 1 mm precision (American Medical do Brazil™, São Paulo, Brazil). Body mass index (BMI) was calculated (kg/m^2^). Waist circumference was taken midway between the lowest rib and the top of the iliac crest. Hip circumference was taken at the widest diameter of the buttocks. The waist-hip ratio was calculated by dividing waist circumference by hip circumference.

#### 2.4.2 Cognitive mental assessment

Cognitive mental status was assessed using the Mini-Mental State Exam (Folstein et al., 1975). Briefly, this consists of a two-part exam, as follows: (i) only vocal responses are required and involve orientation, memory, and attention; and (ii) more complex tasks, such as naming objects, following verbal commands, writing a sentence spontaneously, and copying a polygon. The maximum scores from the first and second sections are 21 and 9, respectively, totaling 30 points.

#### 2.4.3 Functional and strength assessments

The Fugl-Meyer scale was used to assess the degree of motor impairment of participants through abnormal synergic voluntary movements in the motor function domain (Fugl-Meyer et al., 1974). Scale scores range from 0 to 100 points (< 50 points indicates severe motor impairment, 50–84 represents marked impairment, 85–95 is moderate, and 96–99 is slight). Static and dynamic balance were assessed using the Berg Balance scale (Berg et al., 1995), in which a maximal of 56 points could be achieved for items scored on a 0–4 point scale.

15-RM tests were performed to determine the training loads for each resistance exercise, using the standard procedures proposed by the American College of Sports Medicine (ACSM, 2013). The tests were repeated after 30-min to establish the reproducibility of loads. When differences exceeded 5% the test should be repeated after an additional 30-min resting period; however, no participants needed to repeat the test.

#### 2.4.4 Screening and experimental short-term assessments

Hemodynamic variables (i.e., BP, HR, SV, Q, and SVR) and cardiac autonomic control (i.e., HRV indices and BRS) were simultaneously measured by finger photoplethysmography with height correction on the left upper arm (Finometer™, FMS, Amsterdam, Netherlands). Data were downloaded onto a personal computer and analyzed by BeatScope Software (BeatScope 1.1a, Finapres™ Medical Systems, Amsterdam, Netherlands). The BeatScope software performs beat-to-beat analysis of the finger arterial pressure and uses filtering and level correction to calculate reconstructed brachial pressures from finger pressures. The screening visit consisted of a 25 min assessment at rest in the supine position, with data taken from the last 5 min. During the experimental trials, all physiological markers were assessed at baseline for 10 min and during 40 min post-exercise (or CTL) in the laboratory phase.

For spectral analysis of the R-R interval time series, data were processed using a Fast Fourier Transform (FFT) with the Welch’s method and a Hanning window with 50% overlap, using a customized algorithm in the HeartScopeTM II software (version 1.4, A.M.P.S., LLC, NY, United States). Beat-by-beat R-R interval series were then converted into equally spaced time series with 256 ms intervals using cubic spline interpolation (Lazzoli et al., 2003). Spectral analysis was expressed in normalized units (n.u.) (Task-Force, 1996). The ratio between low frequency and high-frequency bands (LF:HF) was used as an index of sympathovagal balance, with the LF band (0.04–0.15 Hz) considered a marker of sympathetic predominance, and the HF band (0.15–0.50 Hz) as a marker of parasympathetic predominance (Cohen and Taylor, 2002). The BRS was analyzed from the alpha index from the low-frequency band (α-LF) of the beat-by-beat SBP and pulse interval (Parati et al., 2000). Only detected spectral gains with coherence >0.5 (arbitrary threshold) were accepted.

#### 2.4.5 Experimental long-term assessments (ambulatory monitoring)

After the laboratory phase, changes in 24-h BP were measured using an automatic noninvasive ambulatory monitor (Spacelabs Medical™ model 90207, Spacelabs Inc., Redmond, WA, United States). The equipment was fitted to each subject on the non-paretic arm at the end of the experimental sessions, using appropriately sized cuffs (see Figure 1). The ABPM fulfilled the criteria of the British Hypertension Society protocol (O'Brien et al., 2000) and was auto-calibrated before each test according to the manufacturer’s instructions. Participants were instructed on how the device worked and to hold their arm as still as possible when activated, proceed with normal activities, and not to shower or exercise until the next morning. Additionally, they were given a standardized activity diary to complete during the 24-h monitoring period, with instructions to log sleep and wake times, and any unusual physical or emotional events.

The monitor was programmed to record BP *via* oscillation every 15 min, except between the hours of 22:00 and 05:00, during which it recorded BP every 30 min to minimize sleep disturbance. The display on the monitor was switched off to prevent feedback. All BP readings rejected by the ABP Report Management System Software (v.2.00.09) (Spacelabs™ Inc., Redmond, WA, United States) as being artifacts (e.g., readings >250 mmHg) were excluded from analyses. Data sets with <80% of BP recordings also were discarded. When participants returned to the laboratory, the data were downloaded to a computer for determination of BP during daytime (12:00–22:00 h), sleeping (22:00–06:00 h), and waking (06:00–12:00 h) hours in CTL and MCT conditions.

Concurrent with 24-h ABPM, a 24-h electrocardiogram R-R interval histogram was obtained using a Cardiolight Cardios digital model with Memory Card, which performed a 3-channel continuous recording subsequently analyzed by the CardioSmart Professional CS 540 program for the removal of artifacts (Cardio System™ Ltda., São Paulo, SP, Brazil). The recording device was fitted at the same location and tests were processed by a single computer, as recommended by the manufacturer. The beat-by-beat R–R interval series were exported and analyzed using CardioSmart™ software (CS-550, Cardios, Brazil). Like ABPM, recordings were averaged for each hour and analyzed for daytime (12:00–22:00 h), sleeping (22:00–06:00 h), and waking (06:00–12:00 h) hours in CTL and MCT conditions.

#### 2.4.6 Mixed circuit training and non-exercise control sessions

The MCT included 10 exercises with 3 sets of 15-RM per exercise, performed using a vertical loading approach. The exercises were chosen to improve functional capacity in typical activities of daily living (e.g., pull and push, sitting and rising, walking up and downstairs) (Billinger et al., 2014) and to enable the reproducibility of the protocol in regular training centers like gyms. For this, the exercises were divided into functional body-weight exercises (i.e., box step-up and squat) and machine-based exercises with external workloads [i.e., leg press, seated row, knee extension, horizontal chest press, knee flexion, shoulder press, hip abduction, and biceps curl (TechnoGym™, Selection Line, Cesena, Italy)], and always performed in this order. A 45-s bout of over ground walking was performed between each exercise, totaling ∼22.5 min of walking throughout the protocol. Participants were instructed to walk at a comfortable self-selected pace. The MCT was preceded by a warm-up consisting of 1 set of 15 repetitions at 50% of 15-RM for the leg press and seated row, with a 45 s walk after each exercise, totaling ∼5 min of warm-up. The CTL consisted of 20 min seated rest and the participants were monitored with the same instruments used in the bouts of MCT. Within 2 min of completing the CTL and bout of MCT, participants were placed in a supine position for a recovery period of 40 min. All participants were familiarized with the exercises included in the MCT before the experimental exercise bouts and had at least 6 months of experience of resistance training. All training bouts were supervised by three experienced exercise professionals to ensure proper exercise technique.

### 2.5 Statistical analyses

All statistical analyses were performed using IBM SPSS Statistics 22 (SPSS™ Inc., Chicago, IL). Data normality was confirmed by the Shapiro-Wilk test, and thus descriptive sample statistics are expressed as mean ± standard deviation (SD). Areas under the curves (AUC) were calculated (Pruessner et al., 2003) for each physiological marker assessed post-MCT/CTL during the 40-min laboratory phase and the 24-h ambulatory phase (split into daytime, sleeping, and waking hours). The AUC is useful for capturing the overall level of BP over time and is considered best practice for the evaluation of BP responses from ABPM (Cook et al., 2004; Nobre and Mion, 2005). Comparisons between MCT and CTL were performed *via* marginal models using the Mixed procedure within SPSS. Compound symmetry was the best-fitting covariance structure for all marginal models, identified as that which minimized Hurvich and Tsai’s criterion value. The normality of the residuals for each statistical model was confirmed using quantile-quantile plots. The marginal model for the LF:HF ratio exhibited heteroscedastic residuals, which were corrected using a natural log transform. A two-tailed *p* value ≤0.05 was accepted as statistically significant.

## 3 Results

No adverse events were reported before, during, or after the MCT bouts. Table 1 describes the participant baseline characteristics, as demographic data, anthropometric assessments, functional motor performance and cognitive mental assessments, resting hemodynamic and cardiac autonomic function assessments, and use of medication.

**TABLE 1:** Characteristics of the study participants.

Variable	Mean ± SD
Sample	7 (3 females)
Age (years)	58 ± 12
Time after stroke (months)	91 ± 55
Anthropometric assessment	
Body mass (kg)	69.2 ± 8.4
Height (cm)	161.9 ± 10.9
Body mass index (kg/m^2^)	26.6 ± 3.7
Waist circumference (cm)	89.5 ± 12.1
Hip circumference (cm)	96.7 ± 11.1
Waist-hip ratio	0.94 ± 0.16
Functional motor performance and cognitive mental assessments	
Fugl-Meyer´s functional scale (0–100)	87 ± 5
Berg´s balance scale (0–56)	55 ± 1
Mini-mental state exam (0–30)	27.2 ± 1.8
Resting hemodynamic and cardiac autonomic function assessments	
Systolic blood pressure (mmHg)	137 ± 14
Diastolic blood pressure (mmHg)	78 ± 13
Mean arterial pressure (mmHg)	100 ± 12
Heart rate (bpm)	61 ± 6
Stroke volume (ml/beat)	89 ± 25
Cardiac output (L/min)	5.5 ± 1.7
Systemic vascular resistance (AU)	1.3 ± 0.5
Low frequency band (n.u.)	43.8 ± 11.7
High frequency band (n.u.)	49.2 ± 7.2
Sympathovagal balance	0.9 ± 0.4
Baroreflex sensitivity (ms × mmHg^−1^)	13.1 ± 5.1
Medication	
Angiotensin-converting enzyme	3
Angiotensin II receptor type 1	1
Diuretic + Angiotensin II receptor type 1 or Angiotensin-converting enzyme	3

### 3.1 Blood pressure

**FIGURE 3:**
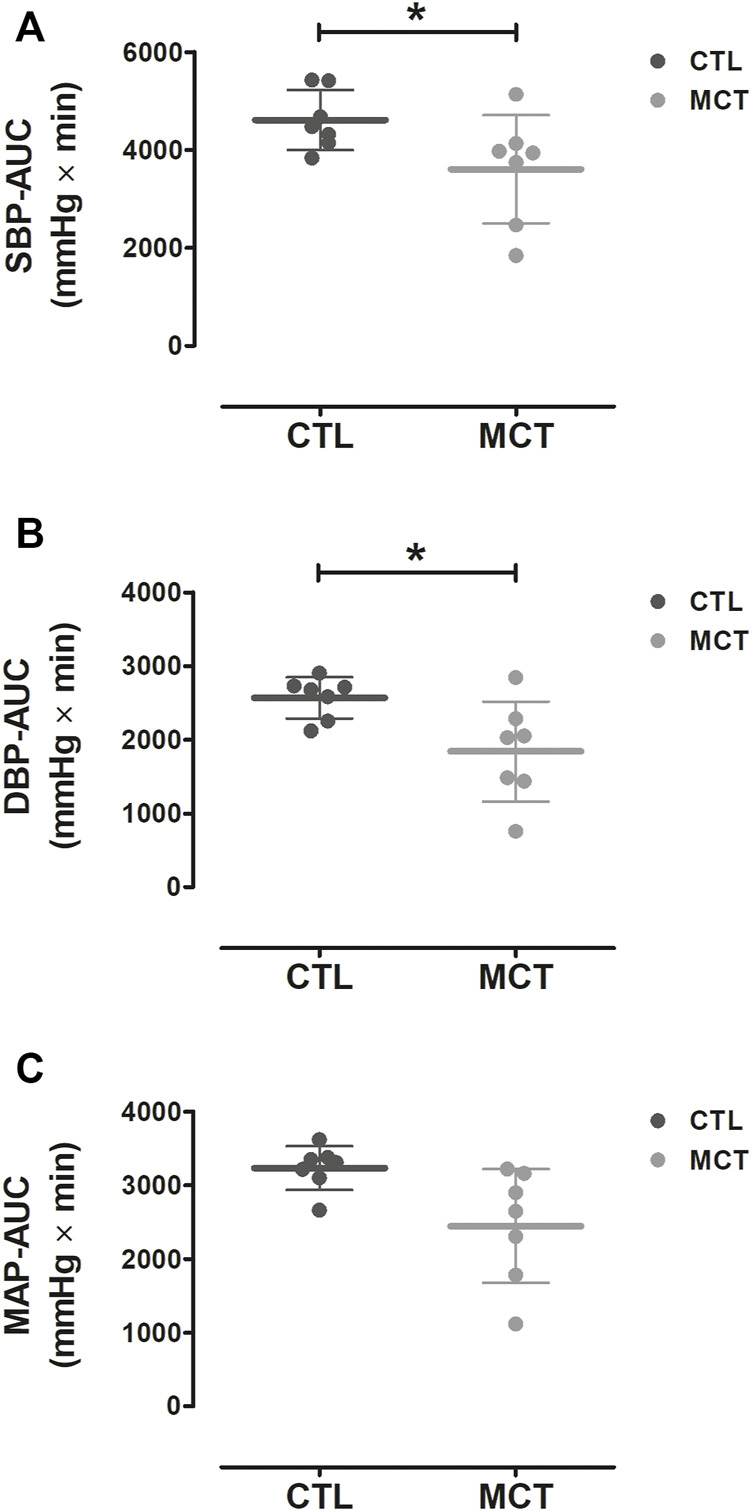
Mean ± SD AUCs for SBP **(A)**, DBP **(B)**, and MAP **(C)** during the first 40 min after the experimental trials. AUC, area under the curve; MCT, mixed circuit training; CTL, non-exercise control session; SBP, systolic blood pressure; DBP, diastolic blood pressure; MAP, mean arterial pressure. *, Significant difference between CTL vs. MCT (*p* < 0.05).

Figures 3A–C shows the AUCs calculated for BP during the first 40-min after the experimental trials. A significant main effect for Condition was observed for SBP [F (1,6) = 6.0, *p* = 0.050] and DBP [F (1,6) = 6.6, *p* = 0.043], whereas no significant difference between conditions were observed for MAP [F (1,6) = 5.5, *p* = 0.058]. SBP and DBP were significantly lower during recovery from MCT compared to CTL [mean diff (CI 95%) = −1008 (−2013 to −1) and −727 (−1422 to −32) mmHg × min, *p* = 0.05 and *p* = 0.043, respectively]. At baseline, no significant differences were observed between Conditions for SBP [F (1,6) = 0.9, *p* = 0.385], DBP [F (1,6) = 0.4, *p* = 0.538], and MAP [F (1,6) = 0.6, *p* = 0.465].

### 3.2 Hemodynamic outcomes

**FIGURE 4:**
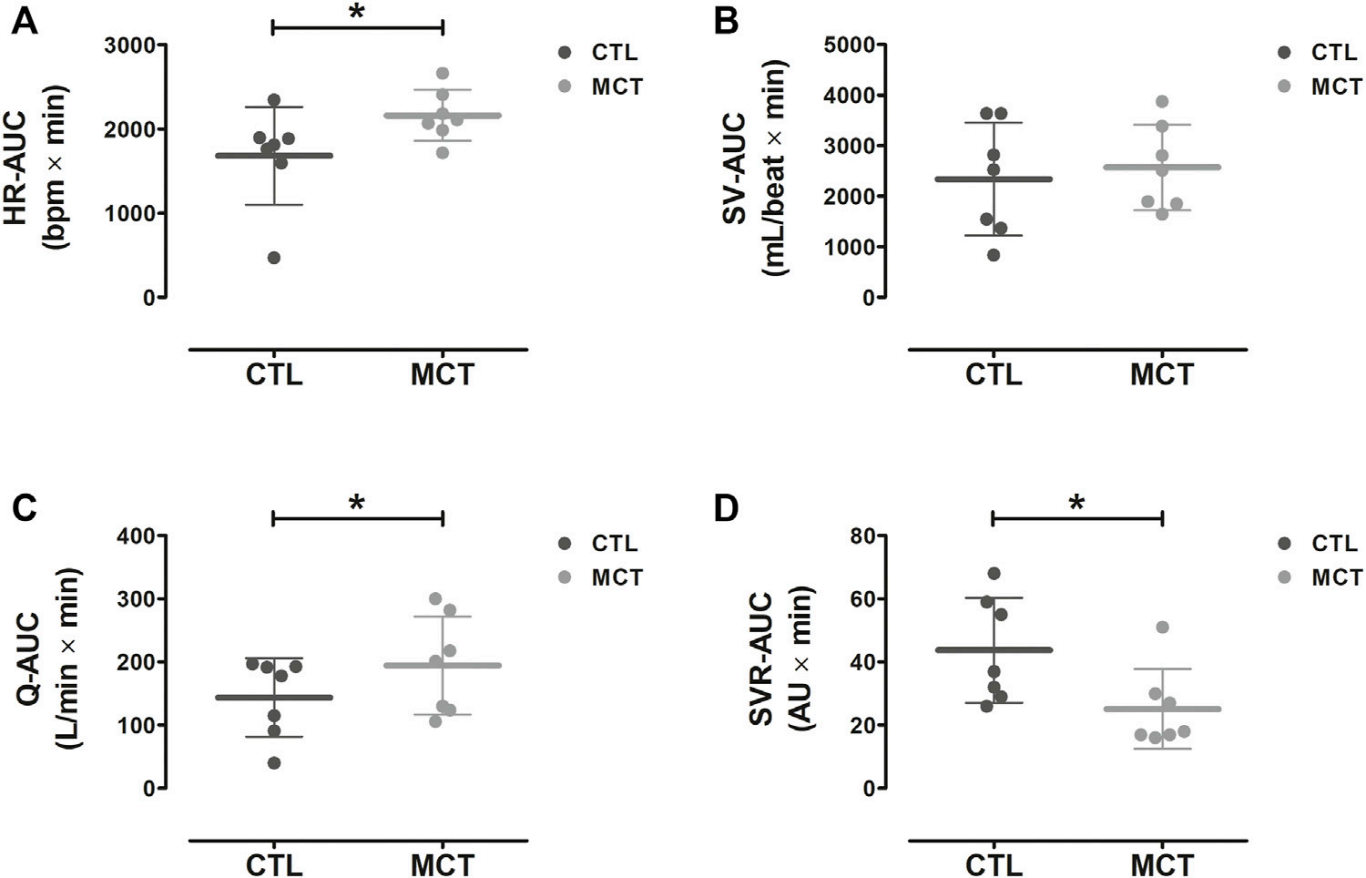
Mean ± SD AUCs for HR **(A)**, SV **(B)**, Q **(C)**, and SVR **(D)** during the first 40 min after the experimental trials. AUC, area under the curve; MCT, mixed circuit training; CTL, non-exercise control session; HR, heart rate; SV, stroke volume; Q, cardiac output; SVR, systemic vascular resistance. *, Significant difference between CTL vs. MCT (*p* < 0.05).

The AUCs calculated for hemodynamic responses during the 40 min of recovery after CTL and MCT are depicted in Figures 4A–D. Significant main effects for Condition were observed for HR [F (1,6) = 7.3, *p* = 0.036] Q [F (1,6) = 10.0, *p* = 0.019], and SVR [F (1,6) = 9.6, *p* = 0.021]. HR and Q were significantly higher (mean diff [CI 95%] = 479 [44 to 914] bpm × min, and 50 [11 to 90] L/min × min, respectively, *p* < 0.05), while SVR was significantly lower during recovery from MCT compared to CTL (mean diff [CI 95%] = −19 [−33 to −4] AU × min, *p* < 0.05). No significant difference in SV was observed between CTL and MCT [F (1,6) = 0.8; *p* = 0.40]. At baseline, no significant differences were observed between Conditions for HR [F (1,6) = 0.1, *p* = 0.883], SV [F (1,6) = 0.7, *p* = 0.429], Q [F (1,6) = 0.4, *p* = 0.541], and SVR [F (1,6) = 0.3, *p* = 0.604].

### 3.3 Cardiac autonomic control

**FIGURE 5:**
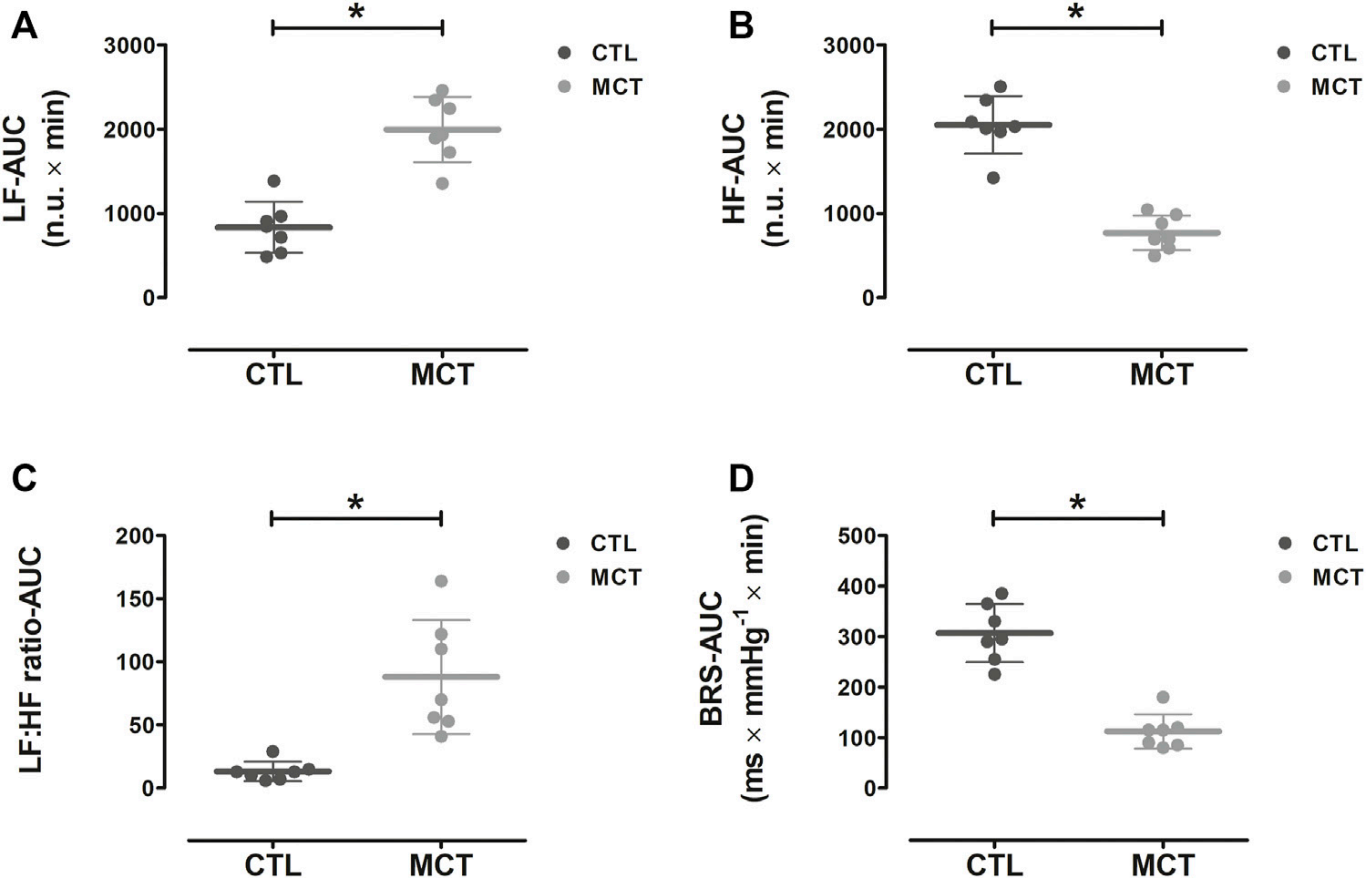
Mean ± SD AUCs for LF **(A)**, HF **(B)**, InLF:HF ratio **(C)**, and BRS **(D)** during the first 40 min after the experimental trials. AUC, area under the curve; MCT, mixed circuit training; CTL, non-exercise control session; LF, low-frequency component; HF, high-frequency component; InLF:HF ratio, logarithmically transformed sympathovagal balance; BRS, baroreflex sensitivity. *, Significant difference between CTL *vs.* MCT (*p* < 0.001).

Figures 5A–D shows the AUCs calculated for autonomic responses after CTL and MCT. Significant differences between Conditions were observed for LF [F (1,6) = 20.8, *p* = 0.004], HF [F (1,6) = 55.9, *p* < 0.001], lnLF:HF ratio [F (1,6) = 26.3, *p* = 0.002], and BRS [F (1,6) = 126.1, *p* < 0.001]. LF and the lnLF:HF ratio were higher in MCT than CTL (mean diff [CI 95%] = 1160 [539 to 1784] n.u. × min, and 0.8 [0.4 to 1.2], respectively, *p* < 0.01). HF and BRS remained significantly attenuated during the post-MCT recovery period (mean diff [CI 95%] = −1282 [−1702 to −863] n.u. × min, and −194 (−237 to −152) ms × mmHg^−1^ × min, respectively, *p* < 0.001). At baseline, no significant differences were observed between Conditions for LF [F (1,6) = 0.1, *p* = 0.790], HF [F (1,6) = 2.3, *p* = 0.173], lnLF:HF ratio [F (1,6) = 2.4, *p* = 0.172], and SBR [F (1,6) = 1.0, *p* = 0.356].

### 3.4 24-h ambulatory blood pressure and cardiac autonomic control

AUCs calculated for ambulatory SBP, DBP, MAP, HR, LF, HF, and LF:HF ratio over the 24-h period after the initial 40-min recovery are shown in Table 2. Significant differences between conditions were observed for SBP [F (1,6) = 9.7, *p* = 0.021], MAP [F (1,6) = 8.2, *p* = 0.029], HR [F (1,6) = 15.9, *p* = 0.007], LF [F (1,6) = 8.2, *p* = 0.029], HF [F (1,6) = 6.7, *p* = 0.041], and lnLF:HF [F (1,6) = 8.8, *p* = 0.025] during daytime (11:00–22:00 h). On average, SBP, MAP, and HF were 7%, 6%, and 26% lower in MCT vs. CTL (*p* < 0.05), whereas HR, LF and lnLF:HF were 4%, 11%, and 13% higher in MCT compared to CTL (*p* < 0.05). There was no main effect for Condition during sleeping and waking hours.

**TABLE 2:** Mean ± SD AUCs calculated for hemodynamic and cardiac autonomic control markers during daytime (12:00–22:00 h), sleeping (22:00–06:00 h), and waking hours (06:00–12:00 h) in the CTL and MCT conditions (*n* = 7).

Variables	Condition		Marginal models		
	CTL	MCT	Mean difference (IC 95%)	F	*p*-value
	Mean ± SD	Mean ± SD			
Day					
SBP (mmHg × min)	1181 ± 104	1092 ± 67	−89 (−157 to −19)	9.7	0.021*
DBP (mmHg × min)	735 ± 98	691 ± 71	−44 (−92 to 4)	5.1	0.065
MAP (mmHg × min)	884 ± 92	826 ± 62	−58 (−106 to −8)	8.2	0.029*
HR (bpm × min)	636 ± 230	659 ± 238	23 (9–36)	15.9	0.007*
LF (n.u. × min)	608 ± 98	675 ± 110	67 (10–124)	8.2	0.029*
HF (n.u. × min)	249 ± 71	185 ± 60	−64 (−124 to −4)	6.7	0.041*
InLF:HF ratio	1.5 ± 0.2	1.7 ± 0.2	0.2 (0–0.3)	8.8	0.025*
Sleep					
SBP (mmHg × min)	870 ± 117	828 ± 114	−42 (−117 to 34)	1.8	0.229
DBP (mmHg × min)	520 ± 101	487 ± 77	−33 (−110 to 44)	1.1	0.338
MAP (mmHg × min)	650 ± 103	593 ± 74	−57 (−117 to 2)	5.6	0.055
HR (bpm × min)	446 ± 44	470 ± 38	23 (−2 to 49)	5.1	0.064
LF (n.u. × min)	474 ± 135	423 ± 121	−51 (−165 to 62)	1.3	0.306
HF (n.u. × min)	226 ± 135	277 ± 110	51 (−62 to 165)	1.2	0.308
InLF:HF ratio	1.4 ± 0.4	1.2 ± 0.4	−0.2 (−0.5 to 0.2)	1.7	0.238
Awake					
SBP (mmHg × min)	602 ± 91	616 ± 58	14 (−67 to 96)	0.2	0.686
DBP (mmHg × min)	375 ± 63	377 ± 50	2 (−64 to 68)	0.1	0.939
MAP (mmHg × min)	449 ± 70	460 ± 49	11 (−62 to 84)	0.1	0.722
HR (bpm × min)	362 ± 34	374 ± 61	12 (−34 to 58)	0.4	0.553
LF (n.u. × min)	350 ± 72	276 ± 82	−74 (−172 to 23)	3.5	0.111
HF (n.u. × min)	135 ± 69	138 ± 61	3 (−72 to 78)	0.0	0.925
InLF:HF ratio	1.3 ± 0.4	1.1 ± 0.3	−0.2 (−0.6 to 0.2)	1.9	0.214

*: Significant difference between CTL vs. MCT.

## 4 Discussion

The present study compared hemodynamics and cardiac autonomic control after MCT and CTL in chronic hemiparetic stroke patients. Although previous studies have investigated the potential of MCT for promoting PEH in individuals with no history of stroke (Brown et al., 1994; Paoli et al., 2013), we unaware of any study that has investigated this in post-stroke patients. To the best of our knowledge, this is the first controlled trial describing cardiovascular responses to acute MCT in this group during a 24-h recovery period. Our data provide original and practically meaningful information, especially considering that impaired autonomic function is common among stroke survivors (McLaren et al., 2005). The main findings were 1) A single bout of MCT was able to induce PEH during the first 10 h of recovery compared to CTL; 2) In the laboratory phase, PEH was concomitant to lowered SVR and attenuated BRS, and increased sympathovagal balance; 3) In the ambulatory phase, PEH during daytime occurred in parallel with increased sympathovagal balance. Overall, these findings suggest that MCT may be an effective antihypertensive therapy for chronic hemiparetic stroke patients, but challenge the role of changes in cardiac autonomic control in eliciting PEH.

### 4.1 The 40-min laboratory phase

The MCT elicited decreases of 22% in SBP and 28% in DBP *vs.* CTL, during the 40 min of passive recovery in the laboratory. Peripheral hemodynamic responses seemed to mediate those reductions, with a 43% reduction in SVR and increases of 34% in Q and 29% in HR compared to CTL. This is in agreement with evidence indicating that in most cases PEH is effectively due to reduced SVR (Brito et al., 2014). Furthermore, we found an increase of 139% in the LF component and a 63% decrease in HF, which is suggestive of sympathetic dominance. Consequently, the sympathovagal balance represented by LF:HF ratio increased (145%). The BRS was blunted after MCT, with a 63% reduction in comparison with CTL.

At least one previous study (Francica et al., 2015) compared BP and cardiac autonomic control after submaximal aerobic exercise in chronic post-stroke women. BP and sympathovagal balance at baseline and immediately post-exercise were consistently higher among stroke patients compared to healthy controls. However, PEH was not detected, as SBP and DBP were almost identical at baseline compared to the 20-min post-exercise recovery period in the stroke patients and healthy controls. Moreover, the LF:HF ratio decreased in both groups *vs.* baseline. These differences in findings between studies were probably due to methodological differences, such as the type of exercise and the associated amount of recruited muscle mass, which may influence acute BP responses (Cunha et al., 2015). While our MCT protocol involved complex tasks incorporating large upper and lower limb muscles (e.g., walking, pushing, pulling, and sitting-and-raising),Francica et al. (2015) applied a submaximal cycle ergometer test involving only lower limbs in a seated position. Finally, post-exercise assessments were very short, being limited to 20-min of recovery in the laboratory *vs.* 40 min in the laboratory phase and 24 h in the ambulatory phase in our study. This does not explain the absence of PEH in their study but does limit the comprehension of the phenomenon from a more ecological perspective. This limitation was addressed in the present study.

Although mixed, the data from previous studies challenge the role of autonomic control as a determinant of PEH in post-stroke patients. In the work by Francica et al. (2015) changes in autonomic modulation were dissociated from BP responses after exercise, while in the present study increases in sympathetic activity and lowered BRS occurred in parallel with a reduction in BP. It has been suggested that fluctuations in autonomic control during PEH might be a consequence rather than a cause of a reduction in BP and that clinically relevant PEH would probably depend on peripheral factors reflecting the ability of vasodilation to counteract the autonomic reaction to bring BP back to baseline levels (i.e., decreased SVR) (Cunha et al., 2016; Fonseca et al., 2018; Farinatti et al., 2021). It is feasible that PEH triggers a rise in sympathovagal balance as a compensatory negative feedback mechanism to a resetting of BRS and reduced BP. A recent meta-analysis (Farinatti et al., 2021) reinforced this premise by investigating the potential relationship between PEH and cardiac autonomic modulation in resistance training. Prolonged reductions in BP of >30 min were inversely related to sympathetic activity, and positively related to parasympathetic activity. Although our results are in agreement with this meta-analysis, caution is needed when making comparisons. Meta-analyzed trials did not include stroke patients and were younger (33.6 ± 15.6 years) than in the present study (58 ± 12 years). Additionally, only 4% of the resistance exercise interventions were performed in a circuit format, highlighting the lack of evidence regarding the effectiveness of this exercise mode in eliciting PEH. Future research is needed to provide insight on how different modes of resistance exercise (i.e., traditional vs. circuit) may impact on the magnitude and duration of PEH, especially in post-stroke patients.

### 4.2 The 24-h ambulatory phase

The 24-h ambulatory monitoring showed that PEH occurred during the daytime (first 10 h of recovery), with reductions of 7% (*p* = 0.021) in SBP and 6% in MAP (*p* = 0.029). An equivalent reduction (6%) was found for DBP, but this effect did not reach statistical significance (*p* = 0.065). Similar to the laboratory phase, reductions in ambulatory BP in the MCT condition were concomitant with greater LF (11%; *p* = 0.029) and attenuated HF (26%; *p* = 0.041), leading to increased sympathovagal balance vs. CLT (*p* = 0.025). It is worth mentioning that patients were medicated with anti-hypertensive drugs (i.e., hypertensive with controlled BP), which are important for stroke management (Rodgers et al., 2004). In a randomized controlled trial involving 6105 patients, anti-hypertensive drug treatment induced an overall mean reduction of 9.0 mmHg (6%) and 5 mmHg (6%) in SBP and DBP, respectively, regardless of baseline BP status (i.e., hypertensive or not) (PROGRESS-Collaborative-Group, 2001). These reductions are similar to the acute BP reductions observed in the present study. The prolonged PEH occurred even though usual medication schedules were maintained, reinforcing the clinical impact of regular exercise for stroke patients.

Lai et al. (2015) investigated the effects of single bouts of aquatic and overground treadmill walking on the magnitude and duration of post-exercise ambulatory BP in post-stroke patients. Exercise bouts were performed for 15 min with an intensity corresponding to 70% VO_2_peak, and post-exercise BP was assessed for 9 h. In comparison to a non-exercise control session, reductions in DBP and SBP of 3%–6% were observed after aquatic but not overground treadmill walking. It is worth noting that the short walking bouts were below the minimum recommended for stroke rehabilitation (Billinger et al., 2014). Given the fact that PEH seems to be dependent on exercise intensity (Halliwill et al., 2013), duration (Halliwill et al., 2013), and volume (Cunha et al., 2016; Fonseca et al., 2018), and those variables were matched, it is feasible to believe that the stimuli from overground treadmill walking was not sufficient to induce post-exercise BP reductions. Although both duration and intensity, and consequently volume, were matched, the mechanisms underlying the PEH were not assessed and the authors could not explain the reasons for the occurrence of PEH only after aquatic but not overground exercise. It was speculated that the aquatic environment could have influenced the sympathetic drive, vascular resistance, or stroke volume, which could explain the occurrence of PEH in only the aquatic condition; but these components were poorly discussed.

Our results showed that PEH occurred during 10 h after MCT, with no significant differences compared to CTL occurring during nighttime (sleep) for both SBP (*p* = 0.229) and DBP (*p* = 0.338). A plausible explanation is nocturnal BP dipping, which may have been superimposed on the exercise-related effects. Reductions of 10%–20% in nocturnal BP are normal and indicate a better cardiovascular prognosis (Yano and Kario, 2012). However, nocturnal BP dipping greater than 20% is exaggerated and may be associated with low blood perfusion in the brain and silent cerebrovascular damage (Kario et al., 1996; Siennicki-Lantz et al., 2007). Participants in the present study exhibited excessive BP nocturnal dipping after both experimental conditions (MCT: SBP −24%; DBP −21% *vs.* CTL: SBP −26%; DPB: −29%), superimposed on the PEH. The pathways underlying these responses are unclear; however, we noticed a more pronounced lnLF:HF dipping after MCT (−29%) *vs.* CTL (−6%). It is therefore possible that changes in sympathovagal balance and BP dipping interact at some level after MCT in chronic stroke patients. Further research is warranted to ratify these findings and address the potential clinical relevance of exercise-related attenuations in nocturnal BP and sympathetic dipping in stroke survivors; however, the literature on BP dipping after exercise in post-stroke patients is scarce. Hence, further research is needed to better elucidate this topic.

### 4.3 Strengths and limitations

This study has strengths that deserve attention. This is the first study to investigate changes in BP and the associated potential mechanistic basis after acute MCT in post-stroke patients, under laboratory (40 min) and 24-h ambulatory conditions, *vs.* a non-exercise control session. These factors increase the external validity of our data. The MCT protocol was consistent with the most recent guidelines for stroke management, including strength and cardiorespiratory components. However, it must be acknowledged that the sample size was small. Even though this is a relatively common issue in studies including post-stroke patients (Francica et al., 2015; Lai et al., 2015), caution is necessary for generalizing the present findings, since low statistical power is associated with an inflated type II error rate (i.e., accepting the null hypothesis when the null hypothesis is false). Also, due to the small sample, it was not possible to perform subgroup analyses according to sex, age, and class of anti-hypertensive drug. Consequently, the present study can be considered as providing important pilot data and further studies with larger samples and addressing the effects of different exercise modalities on acute BP are warranted. Moreover, there is a need for long-term randomized controlled trials to investigate the potential effects of PEH as a determinant of chronic reductions in BP in post-stroke patients.

## 5 Conclusion

A single bout of MCT was capable of eliciting PEH in chronic hemiparetic stroke patients. In the laboratory phase, PEH was concomitant to reduced SVR, increased sympathetic, and decreased parasympathetic modulation. Consequently, reductions in BP in the first 40 min post-exercise occurred in parallel with increased sympathovagal balance and lowered BRS. In the ambulatory phase, SPB and MAP decreased during the daytime (i.e., 10 h after MCT), which was concurrent with increased sympathovagal balance. The PEH was not observed during sleeping hours, probably due to a superimposed effect of nocturnal BP dipping.

## Data Availability

The raw data supporting the conclusion of this article will be made available by the authors, without undue reservation.
